# Age-friendly Neighborhood Environment and Trajectories of Multimorbidity: The Moderating Effect of Life-course Socioeconomic Status

**DOI:** 10.1093/geroni/igaf039

**Published:** 2025-04-23

**Authors:** Jing Liu, Meiteng Yu, Tao Zhang

**Affiliations:** Administrative Office, Shantou University School of Medicine Affiliated Yuebei People’s Hospital, Shaoguan, China; Department of Health Policy and Management, School of Public Administration, Hangzhou Normal University, Hangzhou, China; Department of Health Policy and Management, School of Public Administration, Hangzhou Normal University, Hangzhou, China

**Keywords:** Age-friendly cities and communities, Environmental inequality, Latent growth curve modeling, Longitudinal study

## Abstract

**Background and Objectives:**

Limited longitudinal study exists on the combined effects of environmental factors and life-course socioeconomic status (SES) on multimorbidity in China. This study aims to explore the cumulative impact of age-friendly neighborhoods on multimorbidity over time, focusing on SES moderation.

**Research Design and Methods:**

Analyzing data from 10, 125 participants in the China Health and Retirement Longitudinal Survey (2011–2020), this study assessed multimorbidity through self-reported chronic diseases and evaluated neighborhood environments using 8 domains from the Age-Friendly Cities and Communities framework. Childhood and adulthood SES were measured using latent class analysis. Latent growth curve models examined the effects of neighborhood environment, life-course SES, and their interactions on multimorbidity.

**Results:**

The study found a statistically significant increase in multimorbidity over time, with an intercept of 0.514 and a slope of 0.085. Notably, government support showed an independent association with the baseline number of chronic diseases (*β* = −0.078, *p* < .1). The interaction between government support and childhood SES was also significant (*β* = −0.183, *p* < .05), suggesting that unfavorable childhood SES could reduce the protective effects of government support. Additionally, adulthood SES interacted with factors such as information infrastructure (*β* = −0.068, *p* < .001) and neighborly support (*β* = −0.092, *p* < .1), and transportation interacted with childhood SES (*β* = −0.028, *p* < .05), all negatively affecting the rate of change in multimorbidity. These findings suggest that individuals with higher SES derive greater benefits from these age-friendly neighborhood environment domains compared to their lower SES counterparts.

**Discussion and Implications:**

Age-friendly neighborhoods with strong government support, neighborly support, and developed infrastructure slow multimorbidity progression. However, these benefits are influenced by life-course SES. Policymakers should consider disadvantaged populations’ access to environmental resources and address potential neighborhood socioeconomic health inequalities.

**Translational Significance:**

We identified the cumulative effects of age-friendly neighborhood environments on the trajectories of multimorbidity. Results indicated that the effects of environments slowed down the growing trend of multimorbidity, but these protective health effects differed significantly between socioeconomically advantaged and disadvantaged groups. These findings emphasize environment interventions should consider the fit between environmental resource allocation and individual socioeconomic status, prioritizing the accessibility of high-quality environmental resources for disadvantaged populations, so as to reducing neighborhood socioeconomic inequality in health.

## Background and Objectives

The term “multimorbidity” refers to the coexistence of 2 or more chronic diseases or medical conditions within an individual, often leading to complex healthcare needs and challenges (1). According to comprehensive data, approximately 30%–50% of older adults residing in Western communities experience multimorbidity, significantly affecting their daily lives and health outcomes (2). With the rapid aging of the population in China, it has been estimated that between 2011 and 2015, a staggering 42.4% of individuals aged 50 and older suffered from multimorbidity, highlighting the growing prevalence of this condition in the country (3).

Multimorbidity has emerged as a significant global public health concern, posing a substantial burden on both individuals and governments due to the complex and often intertwined nature of the diseases involved. Many studies have reported that individuals with multimorbidity are at a higher risk of functional decline, such as reduced mobility and cognitive impairment, which can severely limit their ability to perform daily activities (4–7). Furthermore, multimorbidity is associated with reduced quality of life, as individuals may experience pain, discomfort, and limitations in their social and personal activities (5). Premature mortality is also a concern, as individuals with multimorbidity are more likely to experience life-threatening complications and require frequent medical interventions (6). Additionally, the healthcare costs associated with managing multiple chronic conditions can be extremely high, placing a significant financial burden on both individuals and governments (7).

The management and treatment of multimorbidity pose a considerable challenge to the healthcare system, as current service delivery is primarily organized around single diseases, rather than addressing the complex and intertwined nature of multiple conditions (7). Therefore, there is an urgent need to understand the risk factors associated with multimorbidity in order to identify effective prevention strategies. By understanding these risk factors, healthcare providers can better tailor their interventions to meet the unique needs of individuals with multimorbidity, ultimately improving their health outcomes and quality of life.

Theories about social determinants of health highlight that poor health isn’t just caused by personal factors like income, education, job, and social class, or health behaviors. The community environment and other intermediary factors also greatly influence health outcomes (8,9). This principle applies equally to the contributors of multimorbidity. In recent years, a growing number of studies have indicated that larger socioecological contexts in which an individual resides are linked to health behaviors and outcomes (10,11). For example, supportive neighborhood environments can assist older adults’ daily activities and provide more opportunities for social engagements and interactions, thereby reducing the risk of disability and depression (12,13). Disadvantaged neighborhoods with poor economics, violence, disorder, and low social cohesion can cause chronic stress, leading to unhealthy habits like smoking and excessive drinking (14). Improving these neighborhoods could be a key way to reduce multiple health problems.

Among various neighborhood environment improvement initiatives, the Age-Friendly Cities and Communities (AFCC) initiative is a global movement aimed at making urban and rural environments more inclusive, accessible, and supportive of older people (15–17). The core of AFCC framework highlights that an age-friendly environment can facilitate active aging and promote individuals’ wellbeing in their older age through improving the following 8 domains: outdoor spaces and buildings, community support and health services, communication and information, civic participation and employment, respect and social inclusion, social participation, transportation, and housing (15,17). This initiative, often led by the World Health Organization (WHO), has been adopted by various countries to cater to the needs of their aging populations. The common goal is to create environments where older people can live actively, safely, and with dignity.

Over the past decades, scholars have attempted to uncover the relationship between features of age-friendly neighborhood environments and physical and mental health. Their findings revealed that older individuals residing in age-friendly neighborhoods, characterized by supportive housing, adequate transportation facilities, and ample green spaces, exhibited a lower propensity for disability and depression (12,13). Furthermore, residing in advantaged areas during mid-to-late adulthood was found to significantly enhance cognitive function and mitigate its decline, in comparison to one’s childhood environment (18). Two possible reasons can be attributed to this observed correlation. First, the physiological and psychological fragility associated with aging could exacerbate the detrimental effects of adverse neighborhood environments on the health of the older population (19). Additionally, their daily activities being more confined to the neighborhood might increase their exposure duration to potential environmental hazards. Second, the neighborhood environment may harbor a substantial quantity of health-promoting social resources, such as material and emotional support. Given the shrinking social networks of older adults, they tend to rely more heavily on these neighborhood-embedded resources (19,20).

Although growing evidence has shed some light on the potential impacts of an age-friendly neighborhood environment on individual well-being, there are some knowledge gaps that need to be filled. First, residential neighborhoods shape human well-being through compensatory or enabling processes based on prior studies (21). Compensatory processes reflect the function of the environment in maintaining a sense of security and stability for older the, while enabling processes aim to enhance physical functioning and social interactions through environmental support (22,23). Overall, the former focuses on environmental domains meeting older adults’ basic level of needs, and the latter addresses environmental domains supporting older adults’ pursuit of higher-level needs. However, it is unclear which neighborhood environment elements activate compensatory or enabling processes and have more significant impacts on health. Existing studies hardly attempt to investigate the relationship between the level of age-friendliness of places and multiple chronic diseases.

Second, from a life-course perspective, early-life experiences exert formative and enduring influences on long-term health outcomes (24). Empirical evidence demonstrates that prolonged exposure to disadvantaged neighborhood environments during childhood and adolescence significantly elevates the risk of adverse health conditions in adulthood (18,25,26), mediated through pathways such as altered lifestyle behaviors and accelerated development of cardiometabolic risk factors (26). However, existing research on multimorbidity has been limited by cross-sectional designs, which constrain the ability to assess the cumulative effects of environmental exposures over time. To gain a deeper understanding of the long-term effects of environmental exposure, longitudinal data with repeated measurements of multimorbidity are imperative.

Third, the Fundamental Cause Theory further posits that socioeconomic status (SES) serves as a fundamental driver of health disparities. While improvements in environmental resources may confer population-level health benefits, their impact remains inequitably distributed due to differential access and utilization across socioeconomic strata. Higher-SES individuals possess greater capacity to leverage health-promoting resources (27), whereas lower-SES individuals face dual disadvantages: greater exposure to adverse environments (eg, pollution, limited green spaces) and constrained ability to utilize available resources (eg, lower health literacy reducing participation in preventive interventions) (28). Consequently, effective strategies for multimorbidity prevention must address both environmental quality and equitable access to ensure health benefits reach socioeconomically disadvantaged populations (29,30).

The theory of cumulative disadvantage/advantage suggests that childhood SES significantly affects long-term health trajectories (31,32). Higher childhood SES often leads to better access to essential resources like clean water, nutritious food, and safe housing, promoting healthier habits and physical activity (33,34). These early-life advantages can accumulate, enhancing health outcomes and quality of life in adulthood. Conversely, benefits gained in childhood may offset some adverse effects of poor later-life environments. The emergence of multimorbidity arises from a complex mix of environmental exposures (eg, pollution) and lifestyle factors (eg, poor diet, lack of exercise) (35). The corresponding question arises regarding whether the health benefits gained during childhood have the potential to counteract the detrimental effects of subsequent environmental exposures on the occurrence of multimorbidity.

Based on the aforementioned analysis, the following hypotheses are formulated: (a) age-friendly neighborhood environments exhibit cumulative effects on the incidence of multimorbidity; (b) childhood and adulthood SES demonstrates cumulative impacts on multimorbidity; and (c) life-course SES moderates the relationship between neighborhood environments and multimorbidity. To investigate these hypotheses, the current study extended the literature by focusing on the accumulative effects of 8 domains of age-friendly neighborhood environments on trajectories of multimorbidity and the moderating effect of life-course SES on environmental health impact using longitudinal data. Our findings offer important policy implications for the prevention of multiple chronic diseases and the construction of age-friendly neighborhoods in China and other developing countries.

## Research Design and Methods

### Data

Data were drawn from the China Health and Retirement Longitudinal Study (CHARLS), a nationally representative panel survey of individuals aged 45 years and older. This study is dedicated to collecting high-quality data on basic demographic characteristics, family structure, economic support, and health status for Chinese community-dwelling individuals (36). The baseline survey was carried out in 2011, covering 28 provinces, 150 counties/districts, and 450 communities from rural and urban areas, and follow-up surveys were carried out every 2–3 years (36). To date, the CHARLS survey has released 5 waves of data collected from 2011 to 2020. In addition, all living respondents from the first 2 waves (2011 and 2013) were invited to participate in the 2014 CHARLS Life History Survey, aiming to collect information on healthcare history, residential history, education history, and childhood events. The biomedical ethics committee approved this program (IRB00001052-11015), and all participants provided informed consent. Further details can be found on the official website (http://charls.pku.edu.cn)

In this study, we included respondents who completed all 5 longitudinal surveys and the life history survey. Those respondents who were lost to follow-up were excluded (Supplementary Figure 1). Then, we used the neighborhood-level data mainly derived from the baseline CHARLS community survey conducted in 2011 to match with the individual-level dataset. After excluding respondents with missing data on core variables (eg, multimorbidity and SES), a total of 10 125 participants were eligible for analysis.

### Variables

#### Multimorbidity

In the CHARLS, the respondent was asked if they suffer from the following chronic diseases diagnosed by the doctor: high blood pressure, diabetes, cancer, lung disease, heart problems, stroke, psychiatric problems, arthritis, dyslipidemia, liver disease, kidney disease, digestive disease, asthma, and memory problems. Multimorbidity was captured by measuring the number of self-reported physician-diagnosed chronic diseases (0–14) (32).

#### Age-friendly neighborhood environment

Based on the AFCC framework (15,16), we assessed the 8 domains of neighborhood age-friendliness. However, due to not involving some items measuring the domains of “Civic Participation and Employment” and “Respect and Social Inclusion” in the CHARLS, we modified the original AFC framework following the previous studies (12,13). These 2 domains were replaced with “Neighborly support” and “Government support,” respectively. These 2 new dimensions have also been proven to be more appropriate for measuring neighborly environmental friendliness in the context of China (12,13). As a result, the framework of our study contained facilities for health, outdoor spaces and buildings, housing, transportation, facilities for social participation, neighborly support, government support, and infrastructure for communication and information. Details on relative items from the CHARLS community survey and the individual survey mapped onto each domain can be found in Supplementary Table 1. Items’ scores were summed into the total score for each domain for modeling.

#### Life-course socioeconomic status

We constructed separate childhood and adulthood SES indices on multiple measures of socioeconomic conditions in different life course stages. Following previous studies (37,38), childhood SES was assessed by parental education, self-rated childhood economic status, and childhood Hukou. The term “hukou” refers to a household registration system that is unique to China. It serves as a legal document recording an individual’s basic information, such as their official place of residence, and plays a crucial role in determining their access to social services and benefits, including education, healthcare, employment opportunities, and even housing allocations. In terms of adulthood SES, it was assessed by using information from the baseline survey on individual education, occupation, residency, as well as per capita household consumption expenditure. Supplementary Table 2 provides more details on the definition and measurement of these variables.

### Covariates

Demographic characteristics measured by age, gender, and marital status, and health behaviors measured by drinking and smoking were selected as covariates (32). Additionally, we also controlled for self-rated childhood health following the life-course theory. Except for gender and self-rated belonging to the time-invariant variables, the remaining were time-varying.

### Statistical Analyses

A 3-stage analysis strategy was performed in this study. In the first stage, latent class analyses (LCA) were used to define which indicators for childhood (parental education, self-rated childhood economic status, and childhood Hukou) and adulthood SES (individual education, occupation, residency, and per capita household consumption expenditure) naturally cluster together, so as to explore unobserved patterns of SES. This approach allows us to assign respondents to the socioeconomic category they are most likely to belong to, based on their responses to SES indicators (39).

In the second stage, we conducted unconditional latent growth curve models (LGCMs) to determine the initial level and the change rate for the trajectories of multimorbidity. This technology is particularly useful in longitudinal studies where data is collected from the same individuals at multiple time points (40). It allows for estimation of latent variables representing initial levels and rates of change in a particular construct, and to explore how these variables may vary across groups (41). By estimating parameters of intercepts and slopes, the model can describe the trajectories of latent variables over time and identify factors that influence these trajectories. In our study, the model depicted the trajectories of multimorbidity by 2 latent factors: the intercept factor that reflects the average number of chronic diseases at the baseline, and the slope factor that captures the rate of change in the number of chronic diseases over the follow-up period.

**Figure 1.**
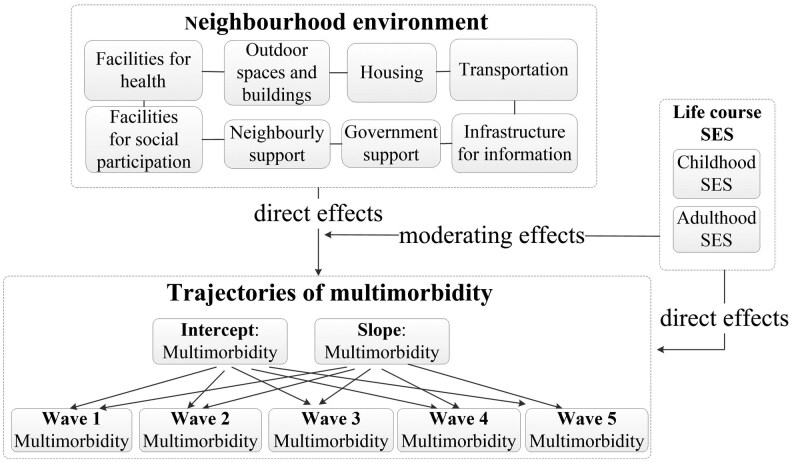
Conceptual framework for the effects of life-course socioeconomic status (SES) and age-friendly neighborhood environment on trajectory of multimorbidity.

In the third stage, the LCA results were subsequently linked to the conditional LGCM to estimate effects of the 8 domains of neighborhood age-friendliness and the class of childhood and adulthood SES on the intercept and slope of multimorbidity, while controlling for all covariates. In order to unravel the moderating effects of childhood and adulthood SES, we added the interaction terms between each domain of age-friendly neighborhood environments and childhood and adulthood SES to the model (12,42). Overall, our model tested the following 3 effects (Figure 1): (a) direct effects of 8 domains of age-friendly neighborhood on trajectories of multimorbidity; (b) direct effects of childhood and adulthood SES on trajectories of multimorbidity; (c) moderating effects of life-course SES on the association of age-friendly neighborhood environment with trajectories of multimorbidity.

All preliminary analyses were conducted in SPSS version 24.0, and all modeling analyses were conducted in Mplus 8.4.

## Results

### Descriptive Analysis

Table 1 presents the basic characteristics of respondents at the baseline survey. Respondents with male and female genders are generally evenly distributed, and the average age was 57.5 years (*SD* = 8.6). 85% of respondents were married, and about one-third of the sample graduated from high school or above. More than half of the respondents work in agriculture (82.9%), reside in rural areas (66.9%), and their parents are illiterate (52.4%). More than 90% of respondents had the agricultural Hukou (93.2%) and did not have a better family economic situation than their neighbors (91.4%) during their childhood.

**Table 1.** Characteristics of Respondents at Baseline Survey

	*N*/mean	%/*SD*
**Gender**		
Male	4 634	45.8
Female	5 491	54.2
**Age**	57.5	8.6
**Marital status**		
Married	8 611	85.0
Others	1 514	15.0
**Education**		
Primary school or illiterate	6 899	68.1
High school or above	3 349	33.1
**Occupation**		
Agricultural work	8 391	82.9
Nonagricultural work	1 734	17.1
**Household consumption expenditure**		
Below the average	6 816	67.3
Equal to or higher than the average	3 309	32.7
**Residency**		
Rural	6 776	66.9
Urban	3 349	33.1
**Parental education**		
Illiterate	5 305	52.4
Nonilliterate	4 820	47.6
**Childhood economic status**		
No better	9 258	91.4
Better	867	8.6
**Type of Hukou**		
Agricultural Hukou	9 438	93.2
Nonagricultural Hukou	687	6.8
**Childhood health status**		
No better	6 592	65.1
Better	3 533	34.9
**Smoking**		
No	6 301	62.2
Yes	3 824	37.8
**Drinking**		
No	6 801	67.2
Yes	3 324	32.8

In terms of multimorbidity, the average value was 1.24 (*SD* = 1.33) at baseline (Supplementary Table 3). There was a significant increase in multimorbidity in the 2013 wave (*p* = .01), 2015 wave (*p* < .001), and 2020 wave (*p* < .001), compared with the 2011 wave.

### Latent Class Analysis

**Figure 2.**
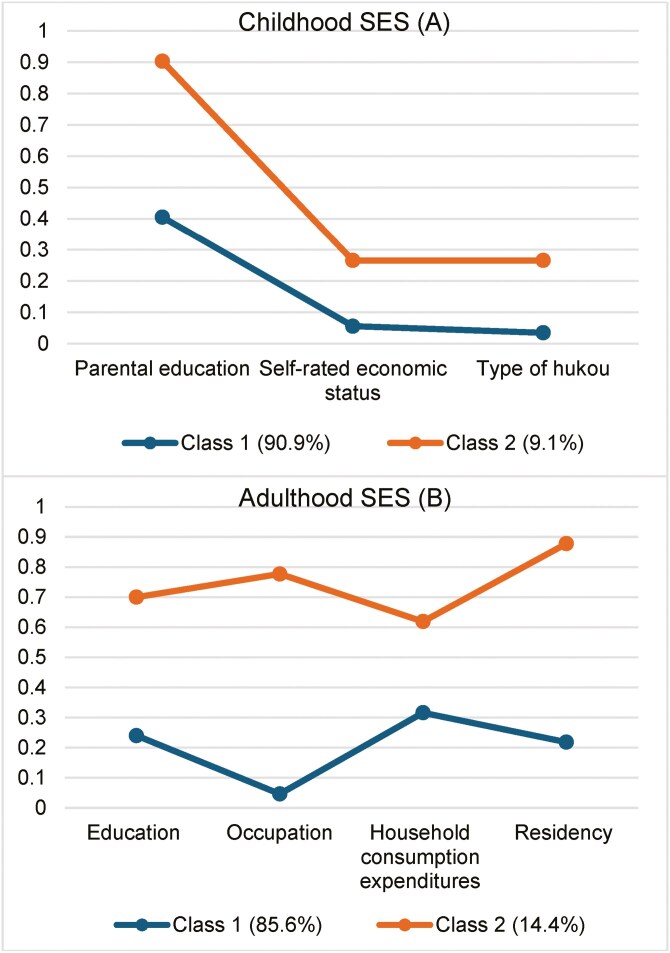
Latent class analysis for patterns of childhood and adulthood socioeconomic status (SES).

Figure 2 shows the LCA results on patterns of childhood and adulthood SES. In this study, we selected the 2-class model based on acceptable model fit and model interpretability (Supplementary Table 4). The vertical axis of Figure 2 indicates the propensity to choose “relatively better” SES categories. Comparing the distribution of 2 classes, respondents in class 1 had a higher possibility of reporting a worse category in each childhood and adulthood SES indicator, and those in class 2 were the opposite. Thus, respondents in class 1 and class 2 were labeled as the “low” and “high” SES groups, respectively. The synthesized childhood and adulthood SES indicators were generated and subsequently linked to modeling.

### The Trajectories of Multimorbidity

We performed linear and nonlinear unconditional LGCMs to depict the trajectories of multimorbidity (Table 2). Model fit indicators showed that the linear model may be more appropriate (root mean square error of approximation <0.06, Comparative Fit Index >0.9, Tucker–Lewis Index >0.9). Results report a positive intercept and slope of multimorbidity, indicating that the multimorbidity of respondents increased significantly over time. Additionally, the variances of intercept and slope revealed remarkable differences between individuals in the initial value and growth rate of multimorbidity.

**Table 2.** Estimations on Trajectories of Multimorbidity Based on Unconditional LGCMs

	Linear Model	Nonlinear Model
**Means**		
Intercept	0.514^*^	0.315^*^
Slope	0.085^*^	0.182^*^
Quadratic term		−0.021^*^
**Variances**		
Intercept	−0.210^***^	−2.542^*^
Slope	−0.108^***^	−3.758^*^
Quadratic term		−0.153^*^
**Goodness-of-fit**		
*χ* ^2^	30 182.453	18 327.108
*p*	<.001	<.001
RMSEA	0.035	0.060
CFI	0.921	0.814
TLI	0.917	0.822

*Notes*: CFI = Comparative Fit Index; RMSEA = root mean square error of approximation; TFI = Tucker–Lewis Index.

^*^ *p* < .05, *** *p *< .001.

### Age-friendly Neighborhood Environment, Life-course SES, and Trajectory of Multimorbidity

Using the multivariate LGCM approach, we estimated the cumulative effects of 8 domains of age-friendly neighborhood environment and life-course SES on the trajectory of multimorbidity, controlling for time-invariant and time-varying covariates (Table 3). Model 1 showed that those who lived in the community with a higher level of transportation (*β* = −0.018, *p* < .05) and infrastructure for information (*β* = −0.046, *p* < .05) were consistently associated with a decreased number of chronic diseases at the baseline. Additionally, adulthood SES (*β* = −0.129, *p* < .001) was found to be negatively associated with the initial level of multimorbidity. In terms of the slope, a high level of facilities for health (*β* = −0.008, *p* < .05), government support (*β* = −0.009, *p* < .05), and infrastructure for information (*β* = −0.020, *p* < .05) might lead to a slower growth rate of multimorbidity. The result of covariates can be found in the Supplementary Table 5.

**Table 3.** LGCM Results of Age-friendly Neighborhood Environment, Life-course SES, and Trajectory of Multimorbidity

	Model 1		Model 2	
	Intercept (*SE*)	Slope (*SE*)	Intercept (*SE*)	Slope (*SE*)
Facilities for health	0.011 (0.007)	−**0.008**^**†**^ **(0.003)**	−0.003 (0.032)	−0.016 (0.012)
Outdoor spaces and buildings	0.003 (0.006)	0.003 (0.002)	−0.023 (0.024)	0.003 (0.009)
Housing	−0.031 (0.018)	0.011 (0.007)	−0.067 (0.100)	−0.019 (0.038)
Transportation	−**0.018**^**†**^ **(0.008)**	0.004 (0.003)	0.088 (0.050)	0.026 (0.017)
Facilities for social participation	−0.004 (0.005)	−0.001 (0.002)	−0.029 (0.022)	−0.001 (0.008)
Neighborly support	0.031 (0.029)	−0.001 (0.011)	−0.044 (0.132)	0.082 (0.051)
Government support	−0.008 (0.010)	−**0.009**^**†**^ **(0.004)**	−0.078^*^ (0.005)	−0.011 (0.019)
Infrastructure for information	−**0.046**^**†**^ **(0.020)**	−**0.020**^**†**^ **(0.008)**	−0.077 (0.082)	0.028 (0.032)
Childhood SES	0.001 (0.026)	0.005 (0.010)	0.247 (0.172)	−0.079 (0.066)
Adulthood SES	−**0.129**^**‡**^ **(0.023)**	0.003 (0.009)	−**0.321**^**†**^ **(0.151)**	−**0.140**^**†**^ **(0.058)**
Facilities for health * Childhood SES			−0.003 (0.026)	0.015 (0.010)
Outdoor spaces and buildings * Childhood SES			0.005 (0.020)	−0.002 (0.008)
Housing * Childhood SES			−0.031 (0.079)	0.030 (0.031)
Transportation * Childhood SES			0.053 (0.035)	−**0.028**^**†**^ **(0.014)**
Facilities for social participation * Childhood SES			0.014 (0.017)	−0.002 (0.007)
Neighborly support * Childhood SES			−0.042 (0.109)	0.020 (0.042)
Government support * Childhood SES			−**0.183**^**†**^ **(0.067)**	0.009 (0.015)
Infrastructure for information * Childhood SES			−0.026 (0.040)	0.032 (0.026)
Facilities for health * Adulthood SES			0.018 (0.022)	−0.009 (0.008)
Outdoor spaces and buildings * Adulthood SES			0.016 (0.016)	0.003 (0.006)
Housing * Adulthood SES			0.066 (0.067)	−0.002 (0.026)
Transportation * Adulthood SES			0.006 (0.032)	0.006 (0.013)
Facilities for social participation * Adulthood SES			0.007 (0.014)	0.002 (0.005)
Neighborly support * Adulthood SES			0.103 (0.093)	−0.092^*^ (0.036)
Government support * Adulthood SES			−0.060 (0.034)	0.008 (0.013)
Infrastructure for information * Adulthood SES			−**0.189**^**‡**^ **(0.053)**	−**0.068**^**‡**^ **(0.020)**
**Goodness-of-fit**				
*χ* ^2^	19 187.373		19 317.258	
*p*	<.001		<.001	
RMSEA	0.012		0.009	
CFI	0.862		0.912	
TFI	0.875		0.944	

*Notes*: CFI = Comparative Fit Index; RMSEA = root mean square error of approximation; TFI = Tucker–Lewis Index.

^*^ *p* < .1. ^†^*p* < .05. ^‡^*p* < .001.

### Interaction Effects of Age-friendly Neighborhood Environment and Life-course SES on Trajectory of Multimorbidity

**Figure 3.**
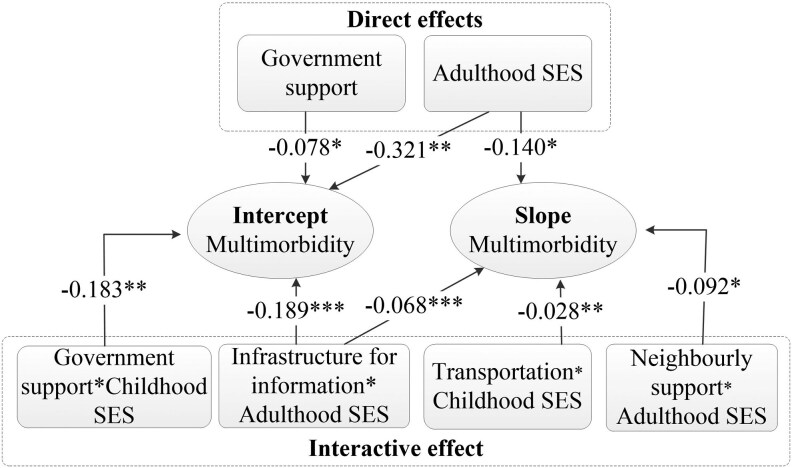
Estimations for direct effects of age-friendly neighborhood environment and life-course socioeconomic status (SES) and their interactive effect on trajectory of multimorbidity. ^*^p < .1, ^**^p < .05, ^***^p < .001.

To investigate the moderating effect of life-course SES, Model 2 incorporated interaction terms between various domains of age-friendly neighborhood environments and childhood and adulthood SES, building upon Model 1 (Table 3). Notably, direct effects were observed for government support and adulthood SES, independent of any interaction effects, as illustrated in Figure 3. Specifically, individuals residing in communities with robust government support exhibited a lower incidence of chronic diseases at baseline (*β* = −0.078, *p* < .1). Furthermore, a higher SES in adulthood caused a reduction in baseline multimorbidity (*β* = −0.321, *p* < .05) and led to a slower progression of multimorbidity over time (*β* = −0.140, *p* < .1).

The results of the moderation analysis revealed significant interaction terms between certain domains of age-friendly neighborhood environments and life-course SES (Figure 3). Specifically, for the baseline level of multimorbidity, we observed a notably negative interaction effect between government support and childhood SES (*β* = −0.183, *p* < .05). A similar significant result was found for the interaction between infrastructure for information and adulthood SES (*β* = −0.189, *p* < .001). These findings indicate that the higher SES throughout an individual’s life course results in fewer chronic diseases at baseline compared to those with lower SES, even when both groups reside in neighborhoods with equivalent levels of government support and information infrastructure. However, it is worth noting that the health-protective benefits of these age-friendly neighborhood environments may be diminished by the adverse impacts of unfavorable childhood and adulthood SES.

Regarding the rate of change in multimorbidity, the interaction terms between infrastructure for information (*β* = −0.068, *p* < .001) and neighborly support (*β* = −0.092, *p* < .1) with adulthood SES, as well as the interaction between transportation and childhood SES (*β* = −0.028, *p* < .05), were significantly negative (Figure 3). These findings imply that these specific domains of neighborhood environments have a more pronounced effect on reducing the growth rate of multimorbidity among individuals with advantageous childhood and adulthood SES. Supplementary Table 6 provides a comprehensive overview of the estimation results for all covariates included in Model 2.

## Discussion and Implications

Using longitudinal data from a large nationally representative cohort in China, this study examined the cumulative effects of 8 domains of age-friendly neighborhood environment at the baseline on the longitudinal trajectories of multimorbidity, with a particular focus on the moderating effects of life-course SES. The findings suggested that the multimorbidity increased significantly over time. The cumulative effects of age-friendly neighborhood environments slowed down this growing trend. However, these protective health effects of environments differed significantly between socioeconomically advantaged and disadvantaged groups.

Among the 8 domains of age-friendly neighborhood environment, government support was found to have independent protective effects on the number of chronic diseases at the baseline. This finding aligns with a previous study that focused on mental health, yielding similar results (12). In this study, government support reflects the top-down approach and policy to build age-friendly neighborhoods through health insurance and pension. Due to the gradual establishment of Universal Health Coverage since 2009 in China, a lack of sufficient financial support hindered access to healthcare before that (43,44). Communities that can provide health insurance for residents clearly lead to timely access to healthcare services, thereby preventing chronic diseases. Similarly, pension insurance is primarily designed to provide income for older adults in China (45). Chronic disease patients covered by pension insurance can receive financial assistance, reducing the household financial burden for treatment. Previous studies have also indicated that the availability of these welfare benefits positively shapes individuals’ health.

More importantly, the interaction of government support with childhood SES was significant, indicating the existence of a moderating effect. Prior studies demonstrated that early-life exposures can have lasting impacts on late-life health (26). Those with low socioeconomic conditions in their childhood were more likely to have been exposed to childhood adversity (eg, childhood abuse and neglect). These early life experiences have been proven to be significantly associated with the occurrence of multimorbidity (32). As a result, the health-protective effect of government support could be reduced even offset by the adverse effect of unfavorable childhood SES. By contrast, individuals with advantaged childhood SES can obtain more health benefits from the government support. This finding underscores the challenge of addressing health inequalities across the life course through equitable allocation of environmental resources.

Similarly, childhood SES also moderated the longitudinal association of transportation with multimorbidity. Prior studies found that convenient transportation can not only help patients access timely medical services but also encourage older adults’ out-of-home behaviors and thus enhance their physiological and psychological functions (46,47). The critical period model assumes that community resources at certain life stages have unique and irreversible effects on later-life health (24). The accumulation of advantage theory also holds similar views (48). It is therefore speculated that being exposed to better transportation conditions seems to exert a lasting impact on reducing the growth rate of multimorbidity over the study period. However, this effect varies across groups experiencing different SES in childhood.

In line with existing evidence (28), adulthood SES has an independent effect, indicating that the experience of upward SES was associated with a reduced risk of being ill. The underlying factors contributing to this observed association are likely mediated by intermediary variables such as lifestyle choices, access to and utilization of health services, as well as neighborhood conditions. Groups with advantageous SES typically benefit more from these intermediary factors, which subsequently contribute positively to health outcomes (28). Additionally, its interaction with infrastructure for information and neighborly support led to a slowing growth rate of multimorbidity. Regarding infrastructures for information, television and Internet actually provide important sources of information and knowledge to prevent the occurrence of chronic diseases (49,50). A high level of neighborly support helps older adults, especially those with functional impairments, to deal with their daily lives easily, but voluntary works in the community promote healthy behavior and lifestyle as well (51). These supportive physical and social environments have been proven to activate the proactivity of community-dwelling residents to perform daily activities independently, do what they value, and maintain health-related behaviors in the long run (14).

Notably, the health protection effects of these 2 neighborhood features appeared to be stronger for those with high adulthood SES. Borrowing from fundamental cause theory (27), persons who are more advantaged in terms of knowledge, money, and beneficial social connections are better positioned to avail themselves of health-promoting resources than less advantaged persons. Predictably, those with advantaged adulthood SES are better at utilizing supportive environmental resources to benefit themselves, so as to result in improved health status. Conversely, those disadvantaged groups seem to have a limited capacity to make full use of high-level environmental resources. For example, health-related information is difficult to understand for people with a low education level. Policy implications from this finding highlight that designing age-friendly neighborhoods should consider the ability of socioeconomically disadvantaged groups to use environmental resources.

### Limitations

Some limitations in this study should be noted. First, due to no follow-up community survey in CHARLS, we only assessed the cumulative impact of neighborhood environment at the baseline on the trajectories of multimorbidity but failed to capture the effects of changes in the neighborhood environments over time. Second, the characteristics of the neighborhood’s built and social environment were assessed through questionnaire survey data. However, it is noteworthy that we lack data that would enable more objective measurements of the physical space conditions within these neighborhoods. Furthermore, our study did not incorporate the actual exposure to the environment, the utilization of environmental resources, or the perceived quality of the neighborhood environment. Third, using 2 types of SES (childhood and adulthood) may be incomplete to reflect life-course SES. Further studies considering more life-stage SES are encouraged. Fourth, the data related to childhood were collected based on self-reports, which may introduce recall bias. Moreover, multimorbidity was assessed via self-reported doctor-diagnosed chronic diseases. Those who do not visit doctors frequently may be unaware of suffering from chronic diseases. Last, it is important to note that, despite employing a cohort study design, this study does not constitute a randomized controlled trial. Consequently, the causal relationship between the neighborhood environment and multimorbidity has not been unequivocally established. There may also be potential for reverse causality between the neighborhood environment and the occurrence of multimorbidity. For example, individuals with a low risk of multimorbidity are more likely to live in age-friendly communities.

## Conclusion

This study revealed the effects of age-friendly neighborhoods on the trajectories of multimorbidity and the moderating effects of life-course SES in China, based on an innovative immediate and cumulative perspective. Our findings suggest that residing in a neighborhood environment characterized by robust government support, high neighborly support, and well-developed information and transportation infrastructure significantly slows the rapid progression of multimorbidity. However, the protective effects are moderated by life-course SES, with higher-SES individuals deriving greater benefits due to their enhanced ability to leverage environmental resources. Importantly, merely improving neighborhood resources may not sufficiently reduce health disparities if structural barriers prevent disadvantaged groups from fully utilizing them. Therefore, interventions must not only ensure equitable access to high-quality environmental resources but also address underlying socioeconomic constraints (eg, health literacy, financial barriers) that limit their effectiveness for vulnerable populations. Effective reduction of health inequalities necessitates simultaneous improvements in environmental quality and individual-level capacities to utilize these enhanced resources.

## Supplementary Material

igaf039_suppl_Supplementary_Materials_1

## Data Availability

This study was preregistered and data are available at https://charls.pku.edu.cn/en/

## References

[1] Diederichs C , Berger K, Bartels DB. The measurement of multiple chronic diseases—a systematic review on existing multimorbidity indices. J Gerontol A Biol Sci Med Sci. 2011;66(3):301–311. https://doi.org/10.1093/gerona/glq208 21112963

[2] Holzer BM , Siebenhuener K, Bopp M, Minder CE. Evidence-based design recommendations for prevalence studies on multimorbidity: Improving comparability of estimates. Popul Health Metr. 2017;15:9. https://doi.org/10.1186/s12963-017-0126-4 28270157 PMC5341353

[3] Yao SS , Cao GY, Han L, et al Prevalence and patterns of multimorbidity in a nationally representative sample of older Chinese: Results from the China Health and Retirement Longitudinal Study. J Gerontol A Biol Sci Med Sci. 2020;75(10):1974–1980. https://doi.org/10.1093/gerona/glz185 31406983

[4] Makovski TT , Schmitz S, Zeegers MP, Stranges S, van den Akker M. Multimorbidity and quality of life: Systematic literature review and meta-analysis. Ageing Res Rev. 2019;53:100903. https://doi.org/10.1016/j.arr.2019.04.005 31048032

[5] Ryan A , Wallace E, O’Hara P, Smith SM. Multimorbidity and functional decline in community-dwelling adults: A systematic review. Health Qual Life Outcomes. 2015;13:1–13. https://doi.org/10.1186/s12955-015-0355-9 26467295 PMC4606907

[6] Nunes BP , Flores TR, Mielke GI, Thumé E, Facchini LA. Multimorbidity and mortality in older adults: A systematic review and meta-analysis. Arch Gerontol Geriatr. 2016;67:130–138. https://doi.org/10.1016/j.archger.2016.07.008 27500661

[7] Wang LL , Si L, Cocker F, Palmer AJ, Sanderson K. A systematic review of cost-of-illness studies of multimorbidity. Appl Health Econ Health Policy. 2018;16(1):15–29. https://doi.org/10.1007/s40258-017-0346-6 28856585

[8] Cockerham WC , Hamby BW, Oates GR. The social determinants of chronic disease. Am J Prev Med. 2017;52(1):S5–S12. https://doi.org/10.1016/j.amepre.2016.09.010 27989293 PMC5328595

[9] Egger G , Dixon J. Beyond obesity and lifestyle: A review of 21st century chronic disease determinants. Biomed Res Int. 2014;2014:1–12. https://doi.org/10.1155/2014/731685 PMC3997940 24804239

[10] Frehlich L , Christie CD, Ronksley PE, Turin TC, Doyle-Baker P, McCormack GR. The neighbourhood built environment and health-related fitness: A narrative systematic review. Int J Behav Nutr Phys Act. 2022;19(1):124. https://doi.org/10.1186/s12966-022-01359-0 36153538 PMC9509561

[11] Frehlich L , Christie C, Ronksley P, Turin TC, Doyle-Baker P, McCormack G. Association between neighborhood built environment and health-related fitness: A systematic review protocol. JBI Evid Synth. 2021;19(9):2350–2358. https://doi.org/10.11124/JBIES-20-00354 33993146

[12] Liu YQ , Pan ZL, Liu Y, Li ZG. Can living in an age-friendly neighbourhood protect older adults’ mental health against functional decline in China? Landsc Urban Plann. 2023;240:104897. https://doi.org/10.1016/j.landurbplan.2023.104897

[13] Pan ZL , Liu YQ, Liu Y, Huo ZW, Han WC. Age-friendly neighbourhood environment, functional abilities and life satisfaction: A longitudinal analysis of older adults in urban China. Soc Sci Med. 2024;340:116403. https://doi.org/10.1016/j.socscimed.2023.116403 37989046

[14] Wang X , Auchincloss AH, Barber S, et al Neighborhood social environment as risk factors to health behavior among African Americans: The Jackson Heart Study. Health Place. 2017;45:199–207. https://doi.org/10.1016/j.healthplace.2017.04.002 28475962 PMC5546244

[15] Rudnicka E , Napierala P, Podfigurna A, Meczekalski B, Smolarczyk R, Grymowicz M. The World Health Organization (WHO) approach to healthy ageing. Maturitas. 2020;139:6–11. https://doi.org/10.1016/j.maturitas.2020.05.018 32747042 PMC7250103

[16] McDonald B , Scharf T, Walsh K. Older people’s lived experience and the World Health Organization age-friendly policy framework: A critical examination of an age-friendly county programme in Ireland. Ageing Soc. 2023;43(8):1784–1809. https://doi.org/10.1017/s0144686x21001355

[17] Rémillard-Boilard S , Buffel T, Phillipson C. Developing age-friendly cities and communities: Eleven case studies from around the World. Int J Environ Res Public Health. 2021;18(1):133–133. https://doi.org/10.3390/ijerph18010133 PMC7795502 33375466

[18] Baranyi G , Conte F, Deary IJ, et al Neighbourhood deprivation across eight decades and late-life cognitive function in the Lothian Birth Cohort 1936: A life-course study. Age Ageing. 2023;52(4):1afad056–1afad012. https://doi.org/10.1093/ageing/afad056 PMC10128164 37097769

[19] Glass TA , Balfour JL. Neighborhoods, Aging, and Functional Limitations. Oxford University Press; 2003.

[20] Zheng ZH , Liu WT, Lu YC, Sun N, Chu YS, Chen H. The influence mechanism of community-built environment on the health of older adults: From the perspective of low-income groups. BMC Geriatr. 2022;22(1):1–12. https://doi.org/10.1186/s12877-022-03278-y 35842581 PMC9288733

[21] Lawton MP. Ageing and the Environment: Theoretical Approaches. Springer; 1982.

[22] Lawton MP. The elderly in context: Perspectives from environmental psychology and gerontology. Environ Behav. 1985;17(4):501–519. https://doi.org/10.1177/0013916585174005

[23] Lawton MP , Brody EM. Assessment of older people: Self-maintaining and instrumental activities of daily living. Gerontologist. 1969;9(3):179–186. https://doi.org/10.1093/geront/9.3_Part_1.179 5349366

[24] Ferraro KF , Shippee TP. Aging and cumulative inequality: How does inequality get under the skin? Gerontologist. 2009;49(3):333–343. https://doi.org/10.1093/geront/gnp034 19377044 PMC2721665

[25] Burdette AM , Needham BL. Neighborhood environment and body mass index trajectories from adolescence to adulthood. J Adolesc Health. 2012;50(1):30–37. https://doi.org/10.1016/j.jadohealth.2011.03.009 22188831

[26] Kivimäki M , Vahtera J, Tabák AG, et al Neighbourhood socioeconomic disadvantage, risk factors, and diabetes from childhood to middle age in the Young Finns Study: A cohort study. Lancet Public Health. 2018;3(8):e365–e373. https://doi.org/10.1016/S2468-2667(18)30111-7 30030110 PMC6079015

[27] Phelan JC , Link BG, Tehranifar P. Social conditions as fundamental causes of health inequalities: Theory, evidence, and policy implications. J Health Soc Behav. 2010;51:S28–S40. https://doi.org/10.1177/0022146510383498 20943581

[28] Pathirana TI , Jackson CA. Socioeconomic status and multimorbidity: A systematic review and meta-analysis. Aust N Z J Public Health. 2018;42(2):186–194. https://doi.org/10.1111/1753-6405.12762 29442409

[29] Craig LS , Cunningham-Myrie CA, Hotchkiss DR, Hernandez JH, Gustat J, Theall KP. Social determinants of multimorbidity in Jamaica: Application of latent class analysis in a cross-sectional study. BMC Public Health. 2021;21(1):1197. https://doi.org/10.1186/s12889-021-11225-6 34162349 PMC8220124

[30] Keats MR , Cui YS, DeClercq V, Grandy SA, Sweeney E, Dummer TJB. Associations between neighborhood walkability, physical activity, and chronic disease in Nova Scotian Adults: An Atlantic PATH Cohort study. Int J Environ Res Public Health. 2020;17(22):8643. https://doi.org/10.3390/ijerph17228643 33233809 PMC7699929

[31] Si YS , Hanewald K, Chen S, Li BQ, Bateman H, Beard JR. Life-course inequalities in intrinsic capacity and healthy ageing, China. Bull World Health Organ. 2023;101(5):307–316C. https://doi.org/10.2471/blt.22.288888 37131938 PMC10140694

[32] Yang L , Hu YY, Silventoinen K, Martikainen P. Childhood adversity and trajectories of multimorbidity in mid-late life: China health and longitudinal retirement study. J Epidemiol Community Health. 2021;75(6):593–600. https://doi.org/10.1136/jech-2020-214633 33293288

[33] Richardson AS , Meyer KA, Howard AG, et al Neighborhood socioeconomic status and food environment: A 20-year longitudinal latent class analysis among CARDIA participants. Health Place. 2014;30:145–153. https://doi.org/10.1016/j.healthplace.2014.08.011 25280107 PMC4252601

[34] Shibalkov IP , Pavlova IA, Nedospasova OP, Tagina EK. Systematization of socioeconomic factors that determine health inequality: A literature review. Tomsk State Univ J. 2021;1(468):101–114. https://doi.org/10.17223/15617793/468/12

[35] Shi WM , Zhang TT, Li YZ, Huang YG, Luo L. Association between household air pollution from solid fuel use and risk of chronic diseases and their multimorbidity among Chinese adults. Environ Int. 2022;170:107635. https://doi.org/10.1016/j.envint.2022.107635 36413929

[36] Chen X , Smith J, Strauss J, Wang Y, Zhao Y. China Health and Retirement Longitudinal Study (CHARLS). Springer; 2017.

[37] Payne CF , Xu KQ. Life course socioeconomic status and healthy longevity in China. Demography. 2022;59(2):629–652. https://doi.org/10.1215/00703370-9830687 35292811

[38] Ye X , Zhu DW, Ding RX, He P. Association of life-course socioeconomic status with allostatic load in Chinese middle-aged and older adults. Geriatr Gerontol Int. 2022;22(5):425–432. https://doi.org/10.1111/ggi.14373 35285137

[39] Porcu M , Giambona F. Introduction to latent class analysis with applications. J Early Adolesc. 2017;37(1):129–158. https://doi.org/10.1177/0272431616648452

[40] Marcoulides KM. Reliability estimation in longitudinal studies using latent growth curve modeling. Meas Interdiscip Res Perspect. 2019;17(2):67–77. https://doi.org/10.1080/15366367.2018.1522169

[41] Lu TY , Poon WY, Tsang YF. Latent growth curve modeling for longitudinal ordinal responses with applications. Comput Stat Data Anal. 2011;55(3):1488–1497. https://doi.org/10.1016/j.csda.2010.10.014

[42] van der Zwan JE , de Vente W, Tolvanen M, et al Longitudinal associations between sleep and anxiety during pregnancy, and the moderating effect of resilience, using parallel process latent growth curve models. Sleep Med. 2017;40:63–68. https://doi.org/10.1016/j.sleep.2017.08.023 29221781

[43] Yu H. Universal health insurance coverage for 1.3 billion people: What accounts for China’s success? Health Policy. 2015;119(9):1145–1152. https://doi.org/10.1016/j.healthpol.2015.07.008 26251322 PMC7114832

[44] Li C , Yu X, Butler JRG, Yiengprugsawan V, Yu M. Moving towards universal health insurance in China: Performance, issues and lessons from Thailand. Soc Sci Med. 2011;73(3):359–366. https://doi.org/10.1016/j.socscimed.2011.06.002 21733610

[45] Liang QH , Chen YX, Zhang Z, An SL. Do the New Rural Pension Scheme promote the health status of chronic patients in old age?—Evidence from CHARLS 2018 in China. BMC Public Health. 2023;23(1):2506. https://doi.org/10.1186/s12889-023-17430-9 38097979 PMC10720147

[46] Nordbakke S , Schwanen T. Transport, unmet activity needs and wellbeing in later life: Exploring the links. Transportation. 2015;42(6):1129–1151. https://doi.org/10.1007/s11116-014-9558-x

[47] Song SQ , Yap W, Hou YT, Yuen B. Neighbourhood built environment, physical activity, and physical health among older adults in Singapore: A simultaneous equations approach. J Transp Health. 2020;18:100881. https://doi.org/10.1016/j.jth.2020.100881

[48] Willson AE , Shuey KM, Glen H, Elder J. Cumulative advantage processes as mechanisms of inequality in life course health. AJS. 2007;112:1886–1924. https://doi.org/10.1086/512712

[49] Jagroep W , Cramm JM, Denktas S, Nieboer AP. Age-friendly neighbourhoods and physical activity of older Surinamese individuals in Rotterdam, the Netherlands. PLoS One. 2022;17(1):e0261998. https://doi.org/10.1371/journal.pone.0261998 35085282 PMC8794150

[50] Nieboer AP , Cramm JM. Age-friendly communities matter for older people’s well-being. J Happiness Stud. 2018;19(8):2405–2420. https://doi.org/10.1007/s10902-017-9923-5

[51] Waverijn G , Heijmans M, Groenewegen PP. Neighbourly support of people with chronic illness; is it related to neighbourhood social capital? Soc Sci Med. 2017;173:110–117. https://doi.org/10.1016/j.socscimed.2016.12.004 27951461

