# Supplementary material for: Age-friendly Neighborhood Environment and Trajectories of Multimorbidity: The Moderating Effect of Life-course Socioeconomic Status
Source: Innov Aging. 2025 Apr 23;9(6):igaf039. doi: 10.1093/geroni/igaf039 (PMC12199366; doi:10.1093/geroni/igaf039)
Supplement: igaf039_suppl_Supplementary_Materials_1 [file igaf039_suppl_supplementary_materials_1.docx]

***Innovation in Aging* Supplementary Material:** **Liu, Yu, and Zhang. Age-friendly neighborhood environment and trajectories of multimorbidity: The** **moderating effect of life-course socioeconomic status.**

**Supplementary Figure 1. Flowchart of the sample selection process**

**Supplementary Table 1. Assessment of** **age-friendly neighborhood environment.**

| **Domain**  **(Total score)** | **Item** | **Mean (SD)** |
| --- | --- | --- |
| **Community facilities and for health**  **(0-6)** | Does the community have outdoor exercise facilities?  (Yes = 1; No = 0) | 2.57  (1.16) |
|  | Does the community have a nursing home?  (Yes = 1; No = 0) |  |
|  | Does the community have a clinic?  (Yes = 1; No = 0) |  |
|  | Does the community have a nearby pharmacy store?  (Yes = 1; No = 0) |  |
|  | Does the community have a public restroom?  (Yes = 1; No = 0) |  |
|  | Does the community have a market/convenience store/grocery?  (Yes = 1; No = 0) |  |
| **Outdoor spaces and buildings**  **(0-5)** | Construction structure of the community^*^  (Organized =1; Disorganized = 0) | 2.83  (1.25) |
|  | Crowdedness of the community^*^  (Sparse =1; Crowded = 0) |  |
|  | Degree of handicapped access of the community^*^  (Convenient =1; No handicapped access = 0) |  |
|  | Tidiness of the roads in the community^*^  (Tidy = 1; Dirty = 0) |  |
|  | Does the community have industrial pollution problem?  (Severe/moderate pollution problem = 0; Otherwise = 1) |  |
| **Housing^**^**  **(0-2)** | Percentage of equipped with indoor barrier-free facilities at home in the community | 1.12  (0.38) |
|  | Percentage of installing elevators for the multi-floor residential buildings in the community |  |
| **Transportation**  **(0-3)** | Is there any bus stop within 1 km of the community?  (Yes = 1; No = 0) | 2.01  (1.01) |
|  | Does the community have roads accessible for cars?  (Yes = 1; No = 0) |  |
|  | What type of road does the community mainly have?  (Paved road = 1; Otherwise = 0) |  |
| **Facilities for social participation**  **(0-6)** | Does the community have room for card games and chess games?  (Yes = 1; No = 0) | 1.54  (1.77) |
|  | Does the community have organizations for helping the older adults and the handicapped?  (Yes = 1; No = 0) |  |
|  | Does the community have an activity centre for older adults?  (Yes = 1; No = 0) |  |
|  | Does the community have an older adult association?  (Yes = 1; No = 0) |  |
|  | Does the community have a dancing team or other exercise organizations?  (Yes = 1; No = 0) |  |
|  | Does the community have an association for calligraphy and painting?  (Yes = 1; No = 0) |  |
| **Neighbourly support^**^**  **(0-4)** | Percentage of taking part in voluntary or charity work in the community | 0.42  (0.24) |
|  | Percentage of caring for the disabled or patients voluntarily in the community |  |
|  | Percentage of interacting with neighbors in the community frequently |  |
|  | Percentage of providing help for neighbors in the community frequently |  |
| **Government support (0-4)** | Percentage of having pension insurance in the community**^++^** | 2.28  (0.72) |
|  | Percentage of having health insurance in the community**^++^** |  |
|  | Does the community provide the minimum living allowance?  (Yes = 1; No = 0) |  |
|  | Does the community provide pensions for older adults aged 65 and above?  (Yes = 1; No = 0) |  |
| **Infrastructure for communication and information^**^**  **(0-3)** | Percentage of having a telephone at home in the community | 1.53  (0.39) |
|  | Percentage of having a TV at home in the community |  |
|  | Percentage of having the internet connection at home in the community |  |

Notes: 1) SD = standard deviation. 2) **^*^** Items from interviewers’ observations from the community survey. 3)**^**^** The domain or item was calculated at the community level using the individual-level data.

**Supplementary Table 2. Variables and measurements.**

| **Variables** | **Measurement** |
| --- | --- |
| ***Outcome*** |  |
| Multimorbidity | A discrete variable indicates the number of respondents suffering from chronic diseases. |
| ***Childhood SES*** |  |
| Parental education | A binary variable indicates that the educational level of the respondents' parents in their childhood; 0= both are illiterate, 1=others |
| Self-rated childhood economic status | A binary variable indicates that indicates whether the respondent's family's economic situation is better than that of their surrounding neighbors; 0=no better, 1=better |
| Childhood Hukou | A binary variable indicates the Hukou type of the respondents during their childhood; 0=agricultural Hukou,1=non-agricultural Hukou |
| ***Adulthood SES*** |  |
| Education | A categorical variable indicating a respondent´s highest level of education; 1= Primary school or illiterate, 2=High school or above |
| Occupation | A binary variable indicates the main occupational type that the respondent is currently or retired from;1=agricultural work, non-agricultural work |
| Per capita household consumption expenditure | A binary variable indicates whether the per capita consumption expenditure of the respondent's household is higher than the average level of their community; 1= below the average, 2= equal to or higher than the average.  The annual household consumption expenses are calculated by adding the following sub items: clothing and bedding, long distance traveling expenses, heating, furniture, consumption of durable goods and electronics, education and training, medical expenditure, fitness expenditures, beauty, automobiles, food, communication fees, transportation, property management fees, taxes and fees and entertainment.  Per capita household consumption expenditure was calculated according to their family size, adjusted by adult equivalence.  The average household consumption level in a community is equal to the total annual consumption of all surveyed households in the community divided by the total number of surveyed households. |
| Residency | A binary variable indicating residence area of respondents; 0=rural,1=urban |
| ***Covariates*** |  |
| Gender | A binary variable scored as 1 for male and 2 for female |
| Age | A discrete variable indicating a respondent´s age |
| Marital status | A binary variable indicating a respondent´s marital status; 1=married, 2=others |
| Smoking | A binary variable indicates whether the respondents have ever smoked; 0=no, 1=yes |
| Drinking | A binary variable indicates that respondents have consumed alcohol in the past year; 0=no, 1=yes |
| Self-rated childhood health | A binary variable indicates whether respondents had better health conditions during childhood compared to their peers; 1=no better, 2=better |

**Supplementary Table 3. The change in multimorbidity over time (2011-2020).**

|  | Mean | SD | p |
| --- | --- | --- | --- |
| 2011 wave (baseline) | 1.24 | 1.33 |  |
| 2013 wave | 1.26 | 0.58 | 0.01 |
| 2015 wave | 1.23 | 1.30 | 0.37 |
| 2018 wave | 2.35 | 1.91 | <0.001 |
| 2020 wave | 2.57 | 0.91 | <0.001 |

**Supplementary Table 4. Latent class analysis model fit.**

|  | Childhood SES | Adulthood SES |
| --- | --- | --- |
| BIC | 24760.900 | 45385.003 |
| p for LMRT | <0.001 | <0.001 |
| P for BLRT | <0.001 | <0.001 |
| Entropy | 0.878 | 0.875 |

**Supplementary Table 5. The impacts of covariates on the trajectory of multimorbidity based on the LGCM without interaction terms.**

|  | Intercept | Slope | 2011 | 2013 | 2015 | 2018 | 2020 |
| --- | --- | --- | --- | --- | --- | --- | --- |
|  | Coef.  (SE) | Coef.  (SE) | Coef.  (SE) | Coef.  (SE) | Coef.  (SE) | Coef.  (SE) | Coef.  (SE) |
| Gender (ref.=male) |  |  |  |  |  |  |  |
| Female | **0.091^***^**  **(0.020)** | 0.005  (0.008) |  |  |  |  |  |
| Self-rated childhood health (ref.=bad) |  |  |  |  |  |  |  |
| Good | **-0.032^***^**  **(0.007)** | **-0.005^**^**  **(0.003)** |  |  |  |  |  |
| Age |  |  | **0.020^***^**  **(0.001)** | 0.001  (0.001) | **0.019^***^**  **(0.001)** | **0.035^***^**  **(0.001)** | **0.007^***^**  **(0.001)** |
| Marital status (ref.=married) |  |  |  |  |  |  |  |
| Others |  |  | -0.047  (0.033) | **0.041^**^**  **(0.021)** | -0.032  (0.038) | 0.013  (0.050) | 0.009  (0.026) |
| Smoking (ref.=no) |  |  |  |  |  |  |  |
| Yes |  |  | **0.061^**^**  **(0.030)** | **0.077^***^**  **(0.017)** | **0.049^*^**  **(0.028)** | **0.089^**^**  **(0.040)** | **0.090^**^**  **(0.027)** |
| Drinking (ref.=no) |  |  |  |  |  |  |  |
| Yes |  |  | **-0.149^***^**  **(0.028)** | -0.011  (0.014) | **-0.144^***^**  **(0.027)** | **-0.268^***^**  **(0.040)** | **-0.040^*^**  **(0.022)** |

^*^ *p* <0.1, ^**^ *p* <0.05, ^***^ *p* <0.001.

**Supplementary Table 6: The impacts of covariates on the trajectory of multimorbidity based on the LGCM with interaction terms.**

|  | Intercept | Slope | 2011 | 2013 | 2015 | 2018 | 2020 |
| --- | --- | --- | --- | --- | --- | --- | --- |
|  | Coef.  (SE) | Coef.  (SE) | Coef.  (SE) | Coef.  (SE) | Coef.  (SE) | Coef.  (SE) | Coef.  (SE) |
| Gender (ref.=male) |  |  |  |  |  |  |  |
| Female | **0.090^***^**  **(0.020)** | 0.005  (0.008) |  |  |  |  |  |
| Self-rated childhood health (ref.=not better) |  |  |  |  |  |  |  |
| Better | **-0.032^***^**  **(0.007)** | **-0.006^**^**  **(0.003)** |  |  |  |  |  |
| Age |  |  | **0.020^***^**  **(0.001)** | 0.001  (0.001) | **0.018^***^**  **(0.001)** | **0.035^***^**  **(0.001)** | **0.006^***^**  **(0.001)** |
| Marital status (ref.=married) |  |  |  |  |  |  |  |
| Others |  |  | -0.044  (0.033) | **0.042^**^**  **(0.021)** | -0.032  (0.038) | 0.013  (0.050) | 0.009  (0.026) |
| Smoking (ref.=no) |  |  |  |  |  |  |  |
| Yes |  |  | **0.058^*^**  **(0.030)** | **0.077^***^**  **(0.017)** | **0.048^*^**  **(0.028)** | **0.088^**^**  **(0.040)** | **0.089^**^**  **(0.027)** |
| Drinking (ref.=no) |  |  |  |  |  |  |  |
| Yes |  |  | **-0.147^***^**  **(0.028)** | -0.010  (0.014) | **-0.144^***^**  **(0.027)** | **-0.269^***^**  **(0.040)** | **-0.040^*^**  **(0.022)** |

^*^ *p* <0.1, ^**^ *p* <0.05, ^***^ *p* <0.001.
